# Practical Observations on the Use of the Rhododendron Chrysanthum in Rheumatism and Gout

**Published:** 1781-04

**Authors:** 


II. *Praktifche Bemerkungen über den Gebrauch der Sibirifchen Schneerofe in gichtkrankheiten,* &c. i. e. *Practical Obfervations on the Ufe of the Rhododendron Chryfanthum in Rheumatifm and Gout. By* Alex. Bern. Kölpin, M. D. *Profeffor of Phyfic at Alten-Stettin.* 8vo. 120 *pages, with a copper plate.* Berlin and Stettin, 1779.

THE plant here fpoken of was firft defcribed by Dr. Gmelin in his *Flora Sibirica,* under the name of *Andromeda foliis ovatis, utrinque venofis, corollis campanulatis, obliquis, longiffimis.* It received its prefent appellation of *Rhododendron Chryfanthum* from Dr. Pallas, who obferves in his travels, that the Tartars on the river Jenifey infufe the leaves of it in a large proportion of water, and ufe it as a kind of medicated tea, which has no fenfible effect unlefs it is made ftrong, for then it affects the head; but that if a few of the branches and leaves are boiled boiled in water in a close vessel, a dark coloured bitter decoction is obtained, which, when taken internally, produces feverish heat, a kind of intoxication, and sometimes even stupor and loss of the senses. At the same time the patient experiences a singular pricking sensation in his limbs or other parts of his body, affected with pains or other complaints. The intoxication soon goes off, leaving behind it neither head-ach nor nausea; and in general, after taking one or two doses the patient finds his pains entirely removed. During the heat produced by the exhibition of this remedy, the patient complains of intense thirst; and if cold water is drank in this state, there ensues a violent but salutary vomiting, especially in complaints of the bowels. The Cossacks use it with great success in cases of rheumatism and painful affections of the joints.

Dr. Pallas having obligingly furnished our author with three pounds of this plant, its effects were tried in fifteen cases. The results of these experiments are fully related in the work before us; and from these it appears, that this new remedy is particularly efficacious in chronic rheumatism, and likewise in the gout; that frequently, though not always, it procures immediate relief, and that when continued for ſome length of time it operates a complete cure.

Dr. Kölpin infuſed two drachms of the tops and leaves of the plant in ten ounces of water. The infuſion was kept twenty-four hours in nearly a boiling heat, and then ſtrained off. Two ounces of this medicine were given for a doſe, and repeated after a few hours. In ſome of the caſes, the proportion of the plant was increaſed to half an ounce. The effects of this remedy in the greater number of the patients were a vomiting and purging, with a partial ſweat over the parts affected. Large doſes were found to produce ſtupor and anxiety. In ſome, the pains grew worſe ſoon after taking the medicine; but this effect was in general ſoon followed by a more remarkable relief.

From his different experiments and obſervations on this ſubject, our author is inclined to think, that in a gouty paroxyſm combined with fever, or in acute rheumatiſm, this medicine ought to be preſcribed with great caution. He ſuppoſes it to act immediately on the nervous ſyſtem, and remarks that, in all the caſes, inſtead of quickening the pulſe, it immediately rendered it weaker and ſlower. The puking and purging which this remedy ſo often excited in arthritic patients, even when they had drank nothing after it, are conſidered by Dr. Kölpin as tending to confirm the opinion of ſome phyſicians, that the cauſe of the gout is to be ſought for in the primæ viæ, or at leaſt, that thoſe parts are particularly affected in that diſeaſe.

In one caſe of venereal rheumatiſm the pains were relieved by this remedy; and Dr. Kölpin even thinks that it might perhaps contribute to the radical cure of that diſorder.

Moſt of the patients complained of a ſenſation of heat and conſtriction in the fauces, after taking the medicine; a proof this that the plant poſſeſſes ſome acrimony, though of a volatile nature, as theſe ſenſations ſoon go off.

In robuſt perſons, we are told, that this remedy operates quickly and with violence; but that on the other hand in weakly and emaciated patients, or in old ſubjects, its effects are ſlow and inconſiderable; ſo that in ſome caſes our author has found it exert its effects not till ſeveral days after it had been taken, and from this circumſtance he is led to caution the practitioner not to be too haſty in augmenting the doſe.

Dr.

Dr. Kölpin obſerves, that violent paſſions of the mind conſtantly hinder the ſalutary effects of the medicine. He adds likewiſe, that the ſame doſe does not always produce the ſame effect, even in the ſame patient. When we have reaſon to ſuſpect a foulneſs of the primæ viæ, he adviſes us to obviate this complaint by gentle purgatives, previous to the exhibition of the remedy in queſtion.

In a caſe of Iſchias the Rhododendron, though continued for ſome length of time, did not completely remove the complaint; but it was afterwards cured by the application of a bliſter according to Cotunnius's method.

In gouty, or perhaps what might rather be called rheumatic pains of the teeth, a decoction of the plant was repeatedly applied externally with the deſired effect.

Theſe are the principal reſults of Dr. Kölpin's experiments, which ſeem to have been conducted with accuracy and judgment, and of courſe entitle the Rhododendron Chryſanthum to the notice of the medical practitioner. On the other hand it may be obſerved, that the trials made with this remedy by Dr. Home, and mentioned in our Journal for January (page 10) were very different in their events from thoſe related in the work before us. It muſt therefore be left to future experience to determine which of the two writers is right in his concluſions on this ſubject.

